# Experiments and Numerical Simulation of N-decane/Ethanol Bi-Component Droplet Evaporation

**DOI:** 10.3390/molecules28052391

**Published:** 2023-03-05

**Authors:** Zhenzhong Zhang, Xuefeng Huang, Jiangrong Xu

**Affiliations:** Institute of Energy, Department of Physics, Hangzhou Dianzi University, Hangzhou 310018, China

**Keywords:** alternative fuels, n-decane, bi-component evaporation, isothermal evaporation, microexplosion

## Abstract

The evaporation characteristics of n-decane-based bi-component or multi-component droplets have been veiled for application in advanced combustion. This paper proposes to experimentally investigate the evaporation of n-decane/ethanol bi-component droplets settled in the convective hot air, and numerically simulate the key parameters affecting the evaporation charactersitics. It was found that the evaporation behavior was interactively affected by the mass fraction of ethanol and the ambient temperature. For mono-component n-decane droplets, the evaporation process included the transient heating (non-isothermal) and steady evaporation (isothermal) stages. In the isothermal stage, the evaporation rate followed *d*^2^-law. The evaporation rate constant linearly increased as the ambient temperature enhanced (573~873 K). For n-decane/ethanol bi-component droplets, at low mass fractions (≤0.2), the isothermal evaporation processes were steady due to the good miscibility between n-decane and ethanol, like mono-component n-decane, whereas at high mass fractions (≥0.4), the evaporation process experienced ultrashort heating and fluctuating evaporation stages. During the fluctuating evaporation, the bubbles formed inside the bi-component droplets and expanded, resulting in the occurrence of the microspray (secondary atomization) and the microexplosion. The evaporation rate constant of bi-component droplets increased as the ambient temperature enhanced, and showed a “V-shaped” trend with the increase of the mass fraction, and the evaporation rate constant was the smallest at 0.4. The evaporation rate constants based on the numerical simulation by using the multiphase flow model and Lee model showed reasonable agreement with the experimental ones, suggesting a potential of application in practical engineering.

## 1. Introduction

N-decane has been considered as a promising substitute for aviation kerosene applied in aircraft engines because of good thermal stability and low saturated vapor pressure, etc. [1]. Dagaut et al. [2,3] indicated that n-decane had a comparable high-pressure oxidation and combustion performance to kerosene. Many studies have also revealed that the binary or trinary blends of n-decane had the potential of utilization in alternative fuels as diesel, gasoline, and kerosene, such as n-decane + n-hexane, n-decane + n-propylbenzene, n-decane + benzene, n-decane + toluene, n-decane + ethylbenzene, n-decane + trimethylbenzene, n-decane + propylcyclohexane [4]. Bioethanol is one of the most commonly used biofuels, mainly as an additive for gasoline [5,6]. Considering the current tense situation of fossil energy, an attempt on the bi-component fuel of n-decane/ethanol was proposed and the evaporation characteristics of the bi-component fuel droplets were investigated in this work.

The evaporation characteristics of mono-component fuel droplets have been intensively studied by many researchers. During isothermal evaporation, the evaporation rate of mono-component fuel droplets is governed by the so-called *d*^2^-law, which says that the square diameter of a droplet decreases linearly with the evaporation time [7,8,9]. For bi-component or multi-component droplets, theoretically, each component evaporates at different rates, owing to their own volatility. The evaporation of each component varies with the time, and the consumption of each component is generally controlled by the mass diffusion in the liquid [10,11,12]. In the earlier incorrect viewpoint, the evaporation of multi-component droplets generally follows the batch distillation in a sequence of gasification controlled only by the volatility differentials among the different components. Randolph et al. [13] conducted the evaporation experiments of freely falling bi-component droplets, and the results demonstrated that the average molar fraction of each volatile component steadily decreased quite substantially, the gasification sequence did not appear like batch distillation, and the evaporation seemed to indicate mixed-mode behavior. Therefore, it can be generally accepted that in the comprehensive effect of volatility differentials, liquid-phase mass diffusion, liquid-phase heat diffusion, gas-phase heat and mass diffusion, and droplet surface regression, liquid-phase mass diffusion is crucial to determine the evaporation behavior of a multi-component droplet. Sazhin et al. [14] established a simplified model for bi-component droplet heating and evaporation; many factors, including droplet convective heat transfer from the ambient gas, the distribution of temperature, mass diffusion inside the droplet, etc., were considered. The numerical results demonstrated the evaporation characteristics of ethanol and acetone, and obtained the relationship between the component mass fraction and the evaporation time. Zhang et al. [15] investigated the roles of liquid-phase mass diffusivity in multi-component droplet gasification, and concluded that the liquid phase mass diffusivity was low, leading to a completely different gasification mechanism, with the volatility differential having a minor, passive role.

In addition, internal bubbling in the multi-component droplets is possibly initiated in the droplet lifetime, and the internal vapor pressure is built up. This is beneficial for the microexplosive event and can enhance secondary atomization effects [11,16,17,18]. Han et al. [11] studied the evaporation characteristics of ethanol/diesel/biodiesel multi-component droplet fuel, which showed that the evaporation process of DE10 (diesel (85%) + ethanol (10%) + biodiesel (5%)) and D100 (diesel) can be divided into instantaneous heating, fluctuating evaporation, and equilibrium evaporation stages, and the lifetime of DE10 is shorter than that of D100. During the fluctuating evaporation, the microexplosion of a droplet can be clearly observed, and the normalized square diameter of droplet fluctuated violently, and the evaporation rate increased.

Although many studies have been carried out to build a simplified model and predict the droplet regression of bi-component droplets, the comparison of experimental and numerical results has been scarcely made, and, thus, the numerical simulation has not been substantially applied in practical engineering. Therefore, this work will focus on the experiments and simulation of the evaporation characteristics of n-decane/ethanol bi-component droplets in laminar high-temperature air, analyze and discuss the influences of the ethanol mass fraction and the ambient temperature on the droplet evaporation characteristics, obtain the droplet evaporation regression rates in the isothermal stage, and make a comparison to the evaporation rate constants between experimental and numerical results.

## 2. Experimental Details

### 2.1. Materials

The n-decane and ethanol were purchased from Macklin Biochemical Co., Ltd., Shanghai, China. The n-decane/ethanol bi-component fuels were prepared by using a two-step method: (1) A certain amount of n-decane and ethanol were successively measured by a measuring cylinder. (2) The ethanol was added into the n-decane and stirred by a glass rod, and then the mixture was vibrated by ultrasonic wave for a few minutes to make them miscible or dispersive.

The ethanol mass fractions were calculated according to the following formula:

(1) $$Y=\frac{\rho_{e}V_{e}}{\rho_{e}V_{e}+\rho_{n}V_{n}}$$

in which *Y* is the mass fraction of ethanol; *ρ_e_*, *ρ_n_* are the densities of the ethanol and n-decane (g/mL); and *V_e_*, *V_n_* represent the volume of the ethanol and n-decane (mL), respectively.

### 2.2. Experimental Setup

The experimental setup was demonstrated in our previous work [9,17], including a hot air supply module, droplet suspension and position module, high-speed imaging and thermal imaging module, and illumination module. The bi-component droplets were formed by squeezing the syringe, and transferred and suspended onto the fiber with a low thermal conductivity coefficient. Then, the droplets were moved into the hot air as-desired temperature by the manipulator with a high position accuracy. The initial diameters of droplets were kept at 0.9 mm or so. The temperature of the hot air can be adjusted from room temperature to 850 °C with a relative uncertainty of less than 2%. The velocity of the hot air was kept at 0.32 m/s, and, thus, the droplets were heated by the heat conduction and convection from the hot air. The droplets will stay at a laminar zone in the hot air. The evaporation processes were recorded by a high-speed camera (Phantom M310, Vision Research Inc., Wayne, NJ, USA) at a frame ratio of 16,000 fps. The surface temperatures of droplets were acquired by an infrared thermal imaging camera ((A655sc, FLIR Systems Inc., Portland, OR, USA) at a frame rate of 200 fps. The high-speed camera and thermal imaging camera were controlled by a synchronizer to keep the time accuracy of sampling time.

### 2.3. Data Processing

To obtain the isothermal evaporation rate constant, the diameters of droplets need to be measured by the digital imaging treatment reported in our previous work [19]. The diameter can be evaluated by the following formula:

(2) $$d=\frac{4S_{d}}{L_{d}}\beta$$

in which *d*, *L_d_*, and *S_d_* are the diameter, perimeter, and area of a droplet, respectively. *β* is the pixel scale factor, which can be calibrated by the microscope graticules.

To eliminate the effect of the initial diameter of droplets on the evaporation in discussion, during the isothermal evaporation, the evaporation rate constant of a single droplet could be normalized as:

(3) $$d^{2}/d_{0}^{2}=1-K_{e}t/d_{0}^{2}$$

in which *d*_0_ is the initial droplet diameter, *t* and *K_e_* represent the evaporation time and the evaporation rate constant, respectively. Thus, the evaporation rate constant can be obtained by the linearly fitting of Equation (3).

## 3. Modeling and Simulation

### 3.1. Model for Bi-Component Droplet Evaporation

For mono- and multi-component droplet heating and evaporation, Sazhin [20] summarized the models based on the analytical solutions to the heat transfer and species diffusion equations. Rybdylova et al. [21,22] demonstrated the results of the implementation of the heating and evaporation model of a mono-component droplet by using ANSYS Fluent and User-Defined Functions (UDF), and compared them with the results of experimental measurements performed at the Combustion Research Facility, Sandia National Laboratories. On the basis of available experimental data and the predictions of the numerical code using the numerical solutions, these models have been validated and verified. Therefore, in this paper, the standard laminar flow model and Lee model were used in the numerical solutions based on ANSYS Fluent, using User-Defined Functions (UDF). The continuity equation, momentum equation, and energy equation that were involved in the multiphase flow model for numerical calculation can be expressed as:

(4) $$\left\{\begin{array}{c} \frac{\partial \left(\alpha_{l}\rho_{l}\right)}{\partial t}+\nabla .\left(\alpha_{l}\rho_{l}{\vec{u}}_{l}\right)=S_{l}\\ \frac{\partial \left(\alpha_{v}\rho_{v}\right)}{\partial t}+\nabla .\left(\alpha_{v}\rho_{v}{\vec{u}}_{v}\right)=-S_{l} \end{array}\right.$$

(5) $$\frac{\partial \left(\alpha_{l}\rho_{l}{\vec{u}}_{l}+\alpha_{v}\rho_{v}{\vec{u}}_{v}\right)}{\partial t}+\nabla \left(\alpha_{l}\rho_{l}{\vec{u}}_{l}{\vec{u}}_{l}+\alpha_{v}\rho_{v}{\vec{u}}_{v}{\vec{u}}_{v}\right)=-\nabla p+\nabla \left(\mu \nabla .\vec{u}\right)$$

(6) $$\frac{\partial \left(\alpha_{l}\rho_{l}E+\alpha_{v}\rho_{v}E\right)}{\partial t}+\nabla \left(\alpha_{l}(\rho_{l}E+p){\vec{u}}_{l}+\alpha_{v}\left(\rho_{v}E+p\right){\vec{u}}_{v}\right)=\nabla .\left(k\nabla T\right)+S$$

in which *l* and *v* represent the liquid-phase and gas-phase. *ρ* represents the density, α represents the volume fraction, $\alpha_{l}+\alpha_{v}=1$. ${\vec{u}}_{l\left(v\right)}$ represents the velocity, $\vec{u}$ represents the average velocity, $\vec{u}=\frac{\alpha_{l}\rho_{l}{\vec{u}}_{l}+\alpha_{v}\rho_{v}{\vec{u}}_{v}}{\alpha_{l}\rho_{l}+\alpha_{v}\rho_{v}}$. ∇p   represents the pressure gradient. $k_{l\left(v\right)}$ represents the thermal conductivity, *k* represents the average thermal conductivity, $k=\alpha_{l}k_{l}+\alpha_{v}k_{v}$. *μ* represents the average viscosity coefficient, $\mu =\alpha_{l}\mu_{l}+\alpha_{v}\mu_{v}$, $\mu_{l\left(v\right)}$ represents the viscosity coefficient. *T* represents the temperature, *t* represents time. *E* represents the total energy,$\;E=e+\frac{1}{2}u^{2}-\frac{p}{\rho }$, *e* represents the enthalpy. *S_l_* represents the mass source term. *S* represents the energy source term, $S=LS_{l}$. *L* is the latent heat of vaporization.

In the existing models, the vapor-liquid phase change model proposed by Lee has been most widely used. For the Lee model, the mass transfer rate from the liquid phase to the gas phase is expressed as [23,24]:

(7) $$S_{l}=coeff\frac{\alpha_{l}\rho_{l}\left(T_{l}-T_{sat}\right)}{T_{sat}},\;(T_{l}>T_{sat})$$

(8) $$\frac{\partial \left(\alpha_{v}\rho_{v}\right)}{\partial t}+\nabla .\left(\alpha_{v}\rho_{v}{\vec{u}}_{v}\right)=S_{l}$$

in which *T_sat_* is the saturation temperature, *coeff* represents the mass-transfer intensity factor with unit s^−1^. For an empirical coefficient, *coeff* is given with different values for solving different problems. In this work, the controllable empirical coefficient *coeff* of 0.6 s^−1^ was adopted.

### 3.2. Simulation for Bi-Component Droplet Evaporation

For the sake of the simplification of the droplet evaporation, it is assumed that the droplet is a symmetrical sphere along the axis and the gravity is not considered. The physical properties for the simulation are listed into Table 1 [25]. The initial droplet diameter and temperature are 0.9 mm and 298 K, respectively. The velocity of the hot air flow is 0.32 m/s, and the hot air temperatures are set to 573 K, 673 K, 773 K, 873 K, respectively.

**Figure 1:**
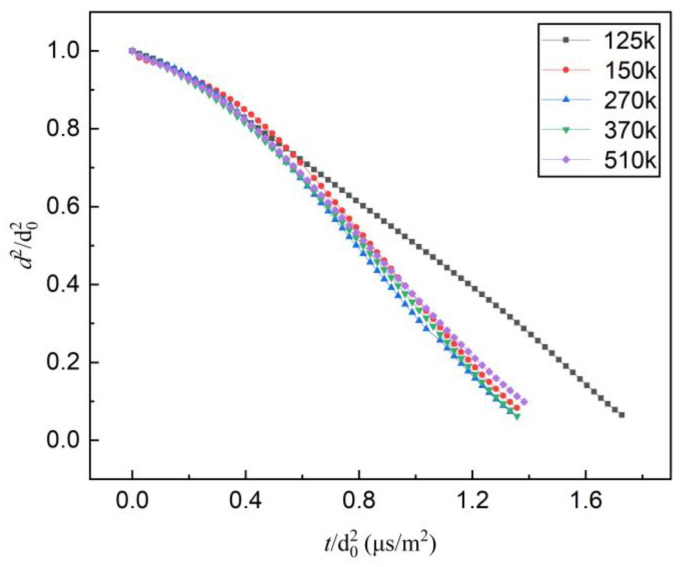
The calculated results at different numbers of grids.

To evaluate the effect of grid numbers for meshing on the calculation, grid independence verification was conducted. The grid numbers of 125,000; 150,000; 270,000; 370,000; and 510,000 were used to calculate the evaporation rate constants of mono-component n-decane at the same operation conditions (in the hot air of 873 K). The results (Figure 1) verified that the grid number has a negligible impact on the calculation, as the numbers of grid are more than 150,000. Therefore, to reduce the CPU time consumption and memory requirements, the grid number of 150,000 was utilized to carry out the calculations for all operation conditions.

**Figure 2:**
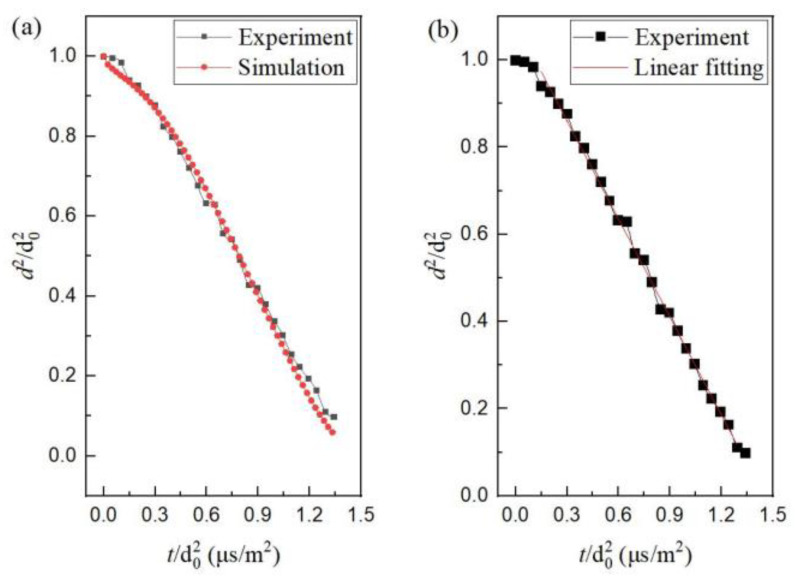
The experimental and calculated curves during transient heating and isothermal evaporation of n-decane; (**a**) the experimental and calculated results; (**b**) the experimental value and linear fitting result.

To verify the reliability of numerical calculation, a comparison of calculation and experiments was made for mono-component n-decane evaporation in the hot air of 873 K. The comparison (Figure 2a) shows that the calculated result kept agreement with the experimental one, including transient heating and isothermal evaporation stages. In the isothermal evaporation stage, it is found that the evaporation follows a so-called *d*^2^-law (Figure 2b). The evaporation rate constant *K_e_* can be obtained by the linear fitting of the experimental and calculated curves shown in Figure 2.

## 4. Results and Discussion

### 4.1. Evaporation of Mono-Component N-decane or Ethanol Droplets

**Figure 3:**
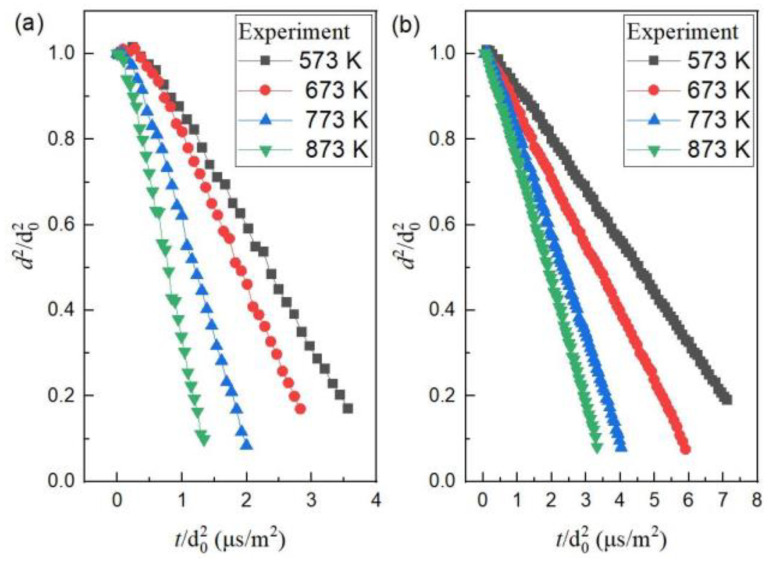
The evaporation curves of mono-component droplets at different ambient temperatures in experiments: (**a**) n-decane; (**b**) ethanol.

In experiments, the evaporation of mono-component n-decane or ethanol droplets suspended in the hot air of different temperatures (Figure 3) demonstrated two stages: Stage I, non-isothermal evaporation (transient heating); Stage II, isothermal evaporation (steady evaporation). During the isothermal evaporation of mono-component n-decane (Figure 3a), the variation of the droplet square diameter was linearly fitted to obtain the slopes. Experiments at each ambient temperature were carried out for about three to five runs. The uncertainty was mainly caused by the random measurement errors for the non-sphericity of the droplets. According to Equation (3), the average evaporation rate constants at different ambient temperatures are listed into Table 2. In a numerical simulation, a similar two-stage evaporation process was demonstrated. The numerical isothermal evaporation rate constants at different ambient temperatures (Table 2) were obtained by linear fitting. By comparison, the numerical results basically keep agreement with the experimental ones, and the relative error is in a reasonable range.

As a droplet is suspended into the convective hot air, the evaporation rate constant of the droplet can be expressed as:

(9) $$K_{e}=\frac{4k_{g}Nu}{\rho_{g}c_{p,g}}\ln\left(B_{T}+1\right),\;B_{T}=\frac{c_{p,g}\left(T_{g}-T_{b}\right)}{L}$$

in which *c_p,g_* and *T_b_* are the specific heat capacity and the boiling point of the droplet, respectively. *B_T_* is the Spalding mass number. *Nu* (Nusselt number) can be expressed as $Nu=2\left[1+Re^{\frac{1}{2}}Pr^{\frac{1}{3}}/3\right]$, in which *Re* and *Pr* represent Reynolds number and Prandtl number, respectively. This shows that the comprehensive physical and heat transfer parameters of droplets, such as the density, dynamic viscosity, thermal conductivity, specific heat capacity, boiling point, latent heat of vaporization, etc., affect the evaporation rate constant [9].

**Figure 4:**
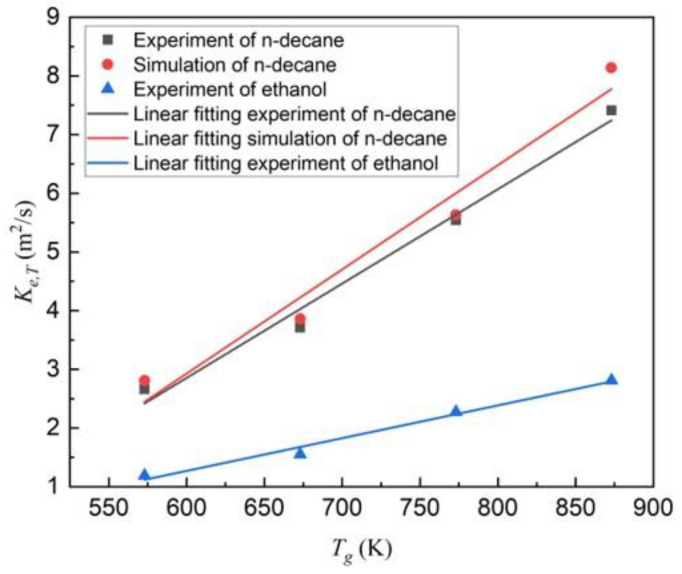
Experimental and numerical evaporation rate constants of mono-component n-decane droplets and experimental evaporation rate constants of mono-component ethanol at different ambient temperatures.

In Equation (9), the surrounding temperature plays a significant role in the evaporation rate constant. In the ambient hot air of 573 K, 673 K, 773 K, and 873 K, the isothermal evaporation rates of mono-component n-decane droplets are plotted in Figure 4. As the ambient temperature enhanced, the evaporation rate constant increased significantly. The relationship between the evaporation rate constant and the ambient temperature (*T_g_*) based on the experiments is followed by:

(10) $$K_{e,T}=\left(0.0161T_{g}-6.796\right)\times 10^{-7}$$

For simulation, the relationship can be expressed as:

(11) $$K_{e,T}=\left(0.0178T_{g}-7.730\right)\times 10^{-7}$$

For the evaporation of mono-component ethanol droplets in experiments, the evaporation rate constants in the ambient hot air of 573 K, 673 K, 773 K, and 873 K were 1.19, 1.55, 2.27, and 2.81 × 10^−7^ m^2^/s, respectively. In comparison to n-decane, the evaporation rates of the ethanol droplets were obviously much smaller at each corresponding ambient temperature, since the latent heat of vaporization of ethanol (8.54 × 10^5^ J/mol) is three times higher than that of n-decane (2.77 × 10^5^ J/mol). The evaporation rate of ethanol also has a linear function with the ambient temperature, i.e., $K_{e,T}=\left(0.00558T_{g}-2.079\right)\times 10^{-7}$.

### 4.2. Evaporation of N-decane/Ethanol Bi-Component Droplets

**Figure 5:**
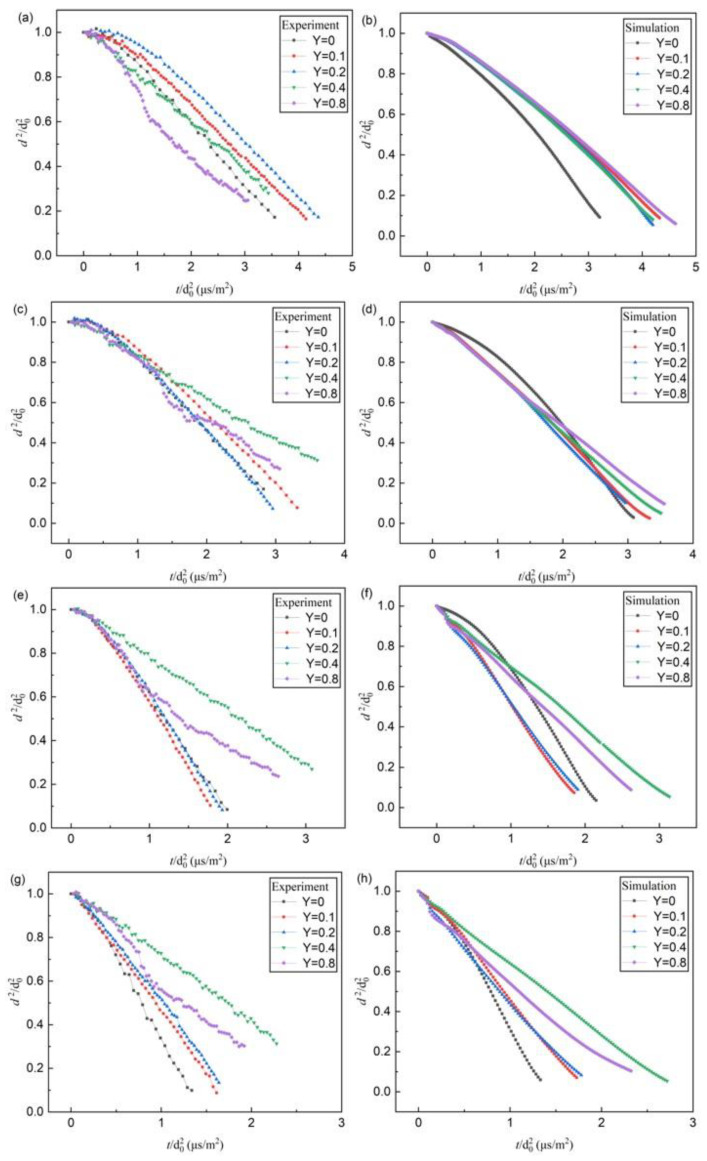
The experimental and numerical evaporation curves of bi-component droplets with different mass fractions of ethanol at different ambient temperatures: (**a**) experimental curves at 573 K; (**b**) numerical curves at 573 K; (**c**) experimental curves at 673 K; (**d**) numerical curves at 673 K; (**e**) experimental curves at 773 K; (**f**) numerical curves at 773 K; (**g**) experimental curves at 873 K; (**h**) numerical curves at 873 K.

Figure 5 shows the experimental and numerical evaporation characteristics of n-decane/ethanol bi-component droplets at different mass fractions of ethanol in the ambient hot air of different temperatures. It was found that the evaporation includes transient heating and isothermal evaporation stages, similar to the evaporation behavior of mono-component n-decane or ethanol droplets. The evaporation of bi-component droplets did not appear in a sequence, such as batch distillation, which seemed to indicate mixed-mode behavior [13]. Generally, ethanol and n-decane have good mutual solubility; therefore, at low mass fractions of 0.1 and 0.2, the isothermal evaporation processes were steady. However, at high mass fractions of 0.4 and 0.8, during the evaporation, the bubbles formed inside the bi-component droplets. The bubbles then expanded as the evaporation evolved, resulting in an enhancement of the droplet diameters. Microspray (secondary atomization) and even microexplosion possibly occurred [11]. After that, the mother droplet diameters became smaller, shortening the evaporation lifetime.

To explore different ethanol mass fractions on the evaporation characteristics of bi-component droplets, the solubility of ethanol in n-decane was considered as a main factor affecting it. The solubility determines whether the bi-component droplet with different ethanol mass fractions can be regarded as a new mono-component-alike fluid. The two cases were classified as Case 1 and Case 2. Case 1: the ethanol mass fraction is less than the saturated concentration, and the mixture of two components is considered as a new mono-component-alike fluid. During the isothermal evaporation, the internal heat and mass transfer of bi-component droplets are homogeneous, and the evaporation follows the *d*^2^-law. The ethanol mass fraction affects the heat and mass transport properties of the bi-component droplets. The more ethanol dissolves into n-decane, the lower the droplet evaporation rate constant is. Case 2: the ethanol mass fraction is higher than the saturated concentration, the mixture can be regarded as an incompatible two-phase fluid, and the excessive ethanol settles the bottom layer of bi-component droplet, owing to the larger density of ethanol. As the mixture evaporates, one part of ethanol evaporates prior to the n-decane due to the lower boiling point of ethanol, and the other part of ethanol possibly dissolves into the n-decane and then evaporates, resulting in a complicated heterogeneous heat and mass transfer inside the bi-component droplets [14]. The influence of the ethanol mass fraction on the evaporation becomes non-monotonous, and the linear *d*^2^-law inadequately responds the evaporation behavior, but it possibly approximately depicts evaporation behavior in the part of isothermal evaporation stage.

For Case 1, the experimental and numerical results show that, as shown in Figure 5 and Table 3, the mass fraction of ethanol is low (≤0.2), and the bi-component droplets generally keep steady evaporation, except for a special case that occurred only once, in which there was abnormal evaporation at the mass fraction of 0.2 and the air temperature of 673 K. The addition of ethanol results in a decrease in the evaporation rate in comparison to the mono-component n-decane, since the addition of ethanol makes the average latent heat coefficient of the droplet increase, and the droplet heat transfer number *B_T_* decrease. According to the linearity of *d*^2^-law, the ethanol completely dissolves in n-decane, and the mixture can be considered as a new mono-component-alike fluid. It can be testified that the evolving curves and evaporation rate constants based on numerical simulation (Figure 5 and Table 3) show a good agreement with the experimental ones in the range of the mass fraction of ≤0.2, suggesting that the numerical simulation has a good suitability for the evaporation of the uniform fluid.

**Figure 6:**
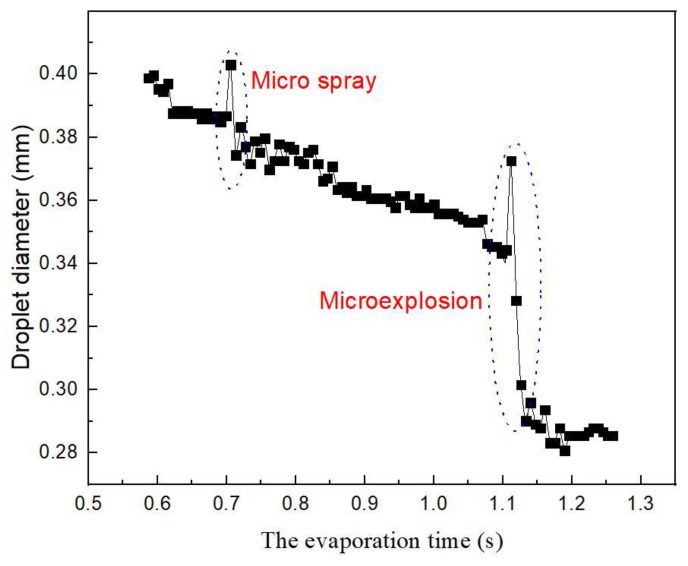
The experimental diameter variation of bi-component droplets with the ethanol mass fraction of 0.8 in the hot air of 573 K.

For Case 2, the mass fraction of ethanol is high (≥0.4). The experimental results demonstrated a fluctuating evaporation, whereas the numerical results show that the evaporation of bi-component droplets is steady in the isothermal stage (Figure 5). The difference is due to the lack of heterogeneous heat and mass transfer inside the bi-component droplet in simulation. In practical experiments, during evaporation, the microspray or microexplosion of droplets can be clearly identified (Figure 6). Since the mass fraction of ethanol added into the n-decane was higher than the saturated concentration, the incompatible two-phase mixture demonstrated the complicated heat and mass transfer process. The excessive ethanol was firstly gasified and formed many bubbles due to the lower boiling of ethanol (351 K) compared with n-decane (447 K). As the bubbles expanded and broke out of the droplet surface, microspray or microexplosion occurred [11]. In the range of the mass fraction of ≥0.4, the normalized square diameters of bi-component droplets violently fluctuated (Figure 5).

Diekmann et al. [26] showed that, at ambient pressure, the critical mole fraction of ethanol in n-decane is 0.5707 in the critical temperature of 257.78 K or 0.5856 (257.72 K) or 0.6201 (257.45 K), and the corresponding solubility of ethanol in n-decane is 30.1% or 31.4% or ~34.5%, respectively. These values stay in the range of 0.2~0.4, which agrees with the results in this work. Therefore, the n-decane/ethanol mixed solution can be regarded as a two-phase fluid, as the ethanol mass fraction exceeds the saturated concentration of ethanol into the n-decane.

In special case the opposite ranking occurs: the droplets with an ethanol mass fraction of 0.2 at the ambient temperature of 673 K evaporate faster than the droplets with 0.1. It is possible that there are two reasons: (1) the mass transfer inside the droplets is complicated, although the evaporation of n-decane/ethanol bi-component droplets with the low mass fractions of ethanol (≤0.2) are steady. Occasionally, we observed the occurrence of droplet diameter burst due to the formation and growth of the bubbles inside the droplets. (2) The evaporation behavior was interactively affected by the mass fraction of ethanol and the ambient temperature. In general, the solubility of ethanol increases with the increase of temperature and decreases with the increase of pressure. 

**Figure 7:**
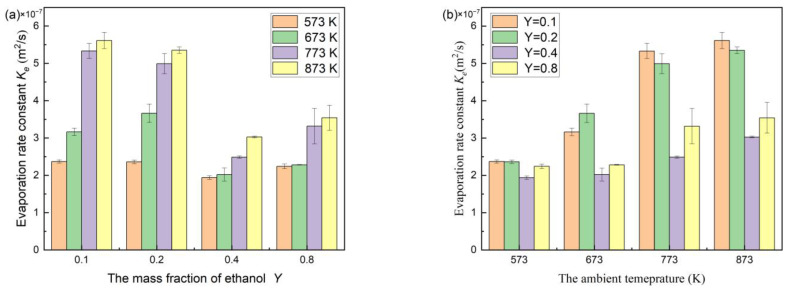
Evaporation rate constants of n-decane/ethanol bi-component droplets: (**a**) at different mass fractions of ethanol at certain a temperature, (**b**) in the ambient hot air of different temperatures at certain a mass fraction.

Besides the mass fraction of ethanol having a significant effect on the evaporation of bi-component droplets, the ambient temperature also largely affects the evaporation behavior. Obviously, at certain a mass fraction of ethanol, as the ambient temperature enhanced, the evaporation rate constants improved (Figure 7a). The improvement of ambient temperature resulted in a reduction of the time of the transient heating stage. When the droplet surface temperature rises to boiling, all the heat absorbed by the droplet is utilized for evaporating. After that, the evaporation of the droplets enters the isothermal stage, the droplet size continuously decreases, the gas-phase component diffusion coefficient *D_v_* becomes greater, and the heat exchange between the droplet surface and the air enhances, and, thus, the evaporation slope becomes larger. 

It is worth noting that the evaporation rate constant of the n-decane/ethanol bi-component droplet is nonlinear to the ambient temperature (Figure 7), unlike the evaporation of mono-component n-decane/ethanol droplets, suggesting that the two factors, the mass fraction and the ambient temperature, are interactive to the evaporation bi-component droplets. At a low ambient temperature of 573 K, the evaporation rates of bi-component droplets with the different ethanol mass fractions were almost equivalent. At 673 K, 773 K, and 873 K, the evaporation rate constant of adding the ethanol of different mass fractions presented “V-shaped” trends (Figure 7b). The evaporation rate stayed the smallest when the mass fraction of ethanol was 0.4.

## 5. Conclusions

The evaporation characteristics of mono-component n-decane and n-decane/ethanol bi-component droplets were experimentally conducted and numerically simulated. In general, the evaporation process experiences non-isothermal and isothermal stages. In the isothermal stage, the evaporation of mono-component n-decane droplets and n-decane/ethanol bi-component droplets with low mass fractions of ethanol (≤0.2) are steady. The unsaturated mixture of mutually soluble n-decane and ethanol can be considered as a new mono-component-alike fluid, and its internal mass transfer during evaporation is homogeneous, and, thus, the evaporation of the new fluid follows classical *d*^2^-law. By contrast, the evaporation of bi-component droplets with high mass fractions of ethanol (≥0.4, higher than the saturated concentration) fluctuates violently and demonstrates microspray or microexplosion, since the excessive ethanol makes the mixture form an incompatible two-phase fluid. One part of dispersive ethanol possibly evaporates, and the other part of dispersive ethanol possibly dissolves into the n-decane and then evaporates, resulting in a complicated heterogeneous mass and heat transfer inside the bi-component droplets.

The numerical simulation based on the multiphase flow model and Lee model well demonstrates the evaporation process, and the calculated steady evaporation rate constants agree well with the experimental ones, especially for mono-component n-decane droplets and n-decane/ethanol bi-component droplets with low ethanol mass fractions. The relative errors between the simulation and experiments for the fluctuating evaporation of bi-component droplets with high ethanol mass fractions are larger, since the simulation neglects the internal complicated heterogeneous heat and mass transfer between the n-decane and ethanol components, owing to the bubble formation. It is vital to consider the internal heat and mass transfer inside non-uniform immiscible two-phase or multi-phase droplets in future work.

According to the experiments and simulation, the ethanol mass fraction and the ambient temperature have significant effects on the evaporation rate constants of bi-component droplets, and interact with the evaporation. The evaporation rate constant increases with increasing ambient temperature, and presents a “V-shaped” trend as the mass fraction of ethanol enhances, and the evaporation rate constant is the smallest at 0.4.

## Figures and Tables

**Table 1:** The physical properties of n-decane and ethanol.

N-decane	
$\rho_{l}\;(\mathrm{kg}/m^{3})$	$724.74-0.8081\times \left(T-300\right)$
$k_{l}\;\left(W/m\cdot K\right)$	$0.1334-0.000237\times \left(T-300\right)$
$c_{l}\;\left(J/\mathrm{kg}\cdot K\right)$	2090
$\mu_{l}\;\left(\mathrm{Pa}\cdot s\right)$	$e^{4.803\times \frac{300}{T}-5.0276}\times 10^{-3}$
$\rho_{v}\;\left(\mathrm{kg}/m^{3}\right)$	0.843
$k_{v}\;\left(W/m\cdot K\right)$	$12.142\times 10^{-3}\times {\left(\frac{T}{300}\right)}^{1.8}$
$c_{v}\;\left(J/\mathrm{kg}\cdot K\right)$	$20.9\times {\left(\frac{T}{300}\right)}^{3}-329.6\times {\left(\frac{T}{300}\right)}^{2}+2013.5\times \left(\frac{T}{300}\right)-47.1$
$\mu_{v}\;\left(\mathrm{Pa}\cdot s\right)$	$\left(0.564+1.75\times 10^{-3}\right)\times \left(T-300\right))\times 10^{-5}$
ethanol	
$\rho_{l}\;\left(\mathrm{kg}/m^{3}\right)$	$766-1.1\times \left(T-333\right)$
$k_{l}\;\left(W/m\cdot K\right)$	0.2468−0.000264×T
$c_{l}\;\left(J/\mathrm{kg}\cdot K\right)$	$\left(1.0264\times 10^{5}-139.63\times T-0.030341\times T^{2}-0.00020386\times T^{3}\right)\times \frac{46}{1000}$
$\mu_{l}\;\left(\mathrm{Pa}\cdot s\right)$	$T^{-3.0418}\times e^{7.875+\frac{781.98}{T}}$
$\rho_{v}\;\left(\mathrm{kg}/m^{3}\right)$	2.06
$k_{v}\;\left(W/m\cdot K\right)$	$-\frac{0.010109\times T^{0.6475}}{1-\frac{7332}{T}-\frac{268000}{T^{2}}}$
$c_{v}\;\left(J/\mathrm{kg}\cdot K\right)$	2407
$\mu_{v}\;\left(\mathrm{Pa}\cdot s\right)$	$\frac{T^{0.8066}}{1+\frac{52.7}{T}}\times 1.0613\times 10^{-7}$

**Table 2:** Experimental and numerical results of mono-component n-decane droplet evaporation in the hot air of different temperatures.

Ambient Temperature	573 K	673 K	773 K	873 K
Experimental evaporation rate (×10^−7^ m^2^/s)	2.66	3.71	5.54	7.41
Numerical evaporation rate (×10^−7^ m^2^/s)	2.81	3.86	5.63	8.14
Relative error	5.6%	4.0%	1.6%	9.9%

**Table 3:** Experimental and numerical simulation results of different mass fractions of ethanol-n-decane droplet evaporation in different gas temperatures.

Ambient Temperature		573 K	673 K	773 K	873 K
0.1	Experimental evaporation rate (×10^−7^ m^2^/s)	2.37	3.16	5.33	5.61
	Numerical evaporation rate (×10^−7^ m^2^/s)	2.33	3.16	5.56	5.82
	Relative error	1.7	0	4.3	3.7
0.2	Experimental evaporation rate (×10^−7^ m^2^/s)	2.36	3.66	4.99	5.35
	Numerical evaporation rate (×10^−7^ m^2^/s)	2.34	3.35	5.05	5.33
	Relative error	0.8	8.5	1.2	0.4
0.4	Experimental evaporation rate (×10^−7^ m^2^/s)	1.94	2.02	2.49	3.03
	Numerical evaporation rate (×10^−7^ m^2^/s)	2.41	2.86	3.07	3.50
	Relative error	24.2	41.6	23.3	15.5
0.8	Experimental evaporation rate (×10^−7^ m^2^/s)	2.24	2.28	3.32	3.54
	Numerical evaporation rate (×10^−7^ m^2^/s)	2.20	2.60	3.48	3.89
	Relative error	1.8	14.0	4.8	9.9

## Data Availability

The data presented in this study are available on request from the corresponding author.

## Nomenclature

Item	Value
*B_M_*	Spalding mass transfer number
*B_T_*	The heat transfer number
*c_p_*	The specific heat capacity
*coeff*	The mass-transfer intensity factor
*D*	The mass diffusion coefficient
*d*	Droplet diameter
*E*	The total energy
*e*	Enthalpy
*h*	Convective heat transfer coefficient
*L*	The latent heat coefficient about evaporation
*L_d_*	The perimeter of a droplet
*K_e_*_,*T*_	The evaporation rate constant
*k*	The thermal conductivity
*Nu*	The Nusselt number
*Pr*	The Prandtl number
*p*	Pressure
*Re*	The Reynolds number
*S*	The energy source term
*S_d_*	Area of a droplet
*S_l_*	The mass source term
*t*	Time
*T*	The temperature
*u*	The average velocity
*V_e_*	The volume of the ethanol
*V_n_*	The volume of the n-decane
*Y*	The ethanol mass fraction
Greek symbols	
*ρ*	The density
*α*	The volume fraction
*β*	The pixel scale factor
*μ*	The average dynamic viscosity coefficient
Subscripts	
*b*	Boiling
e	Ethanol
*g*	Gas-phase
*l*	Liquid-phase
*n*	n-decane
*Sat*	Saturation
*s*	Surface
∞	At infinity space
0	Initial
